# Deep sedation for nasal septal surgery: an observational retrospective study with an inverse probability weighting model

**DOI:** 10.1186/s44158-023-00120-8

**Published:** 2023-09-15

**Authors:** Laura Campiglia, Guglielmo Consales, Lucia Zamidei, Matteo Garotta, Antonio Sarno, Iacopo Cappellini

**Affiliations:** 1 Department of Critical Care, Section of Anesthesiology and Critical Care, Azienda USL Toscana Centro, Ospedale Santo Stefano, Prato, Italy; 2 Department of Surgery, Section of Ear Nose Throat Surgery, Azienda USL Toscana Centro, Ospedale Santo Stefano, Prato, Italy

**Keywords:** Nasal septal surgery, Deep sedation anesthesia, Inverse probability weighting

## Abstract

**Background:**

Septoplasty, a common surgical procedure to correct a deviated septum, can be performed under either general anesthesia or deep sedation anesthesia. The choice of anesthesia can influence the duration of anesthesia and surgical outcomes, impacting the feasibility of outpatient procedures.

**Methods:**

The institutional review board approved the protocol, and we obtained written informed consent from all participants. This retrospective, single-center observational study analyzed data from 586 patients who underwent rhino septoplasty at Santo Stefano Hospital in Prato, Italy, from 2017 to 2021. Patients received either general anesthesia or deep sedation anesthesia. Propensity score matching and inverse probability weighting were used to balance patient characteristics. The main outcome variable was discharge time, with anesthesia time and surgical time as covariates. Statistical analysis was conducted using R software.

**Results:**

Patients who received deep sedation anesthesia had a significantly shorter duration of anesthesia compared to those who received general anesthesia. A multivariate linear regression model showed that the type of anesthesia had a strong positive association with discharge time, while anesthesia time had a weaker negative association, although not statistically significant.

**Conclusions:**

Deep sedation anesthesia is associated with a shorter duration of anesthesia compared to general anesthesia during nasal septal surgery, suggesting it could be a more feasible option for outpatient procedures. However, the choice of anesthesia should be tailored to individual patient factors and surgical requirements. Further research is needed to confirm these findings and explore the potential benefits of sedation anesthesia in outpatient nasal septal surgery.

**Question:**

How do general anesthesia and deep sedation anesthesia compare in terms of duration of anesthesia and surgical outcomes during nasal septal surgery?

**Findings:**

Our study found that deep sedation anesthesia was associated with a shorter duration of anesthesia compared to general anesthesia in patients undergoing nasal septal surgery. However, there were no significant differences in the duration of the surgical procedure.

**Meaning:**

The findings suggest that deep sedation anesthesia could potentially make nasal septal surgery more feasible as an outpatient procedure.

## Introduction

Nasal septal surgery, also known as septoplasty, is a procedure performed to straighten a deviated septum, which is the bone and cartilage dividing the two nostrils. This surgery can be performed under general anesthesia or deep sedation anesthesia, each with their own advantages and drawbacks [1].

General anesthesia (GA), which includes both inhalatory and total intravenous anesthesia (TIVA), is a method that renders the patient completely unconscious during the procedure. It is frequently chosen for septoplasty, ensuring that the patient remains unaware and does not experience any pain during the surgery. The primary advantage of general anesthesia lies in its ability to provide a deeper level of unconsciousness and more precise control over the patient’s vital signs during the procedure. However, despite its widespread use, general anesthesia may have certain potential drawbacks. These can encompass a longer recovery time and, in some cases, an increased risk of complications compared to sedation anesthesia. It is crucial to highlight that these risks can significantly vary based on individual patient factors and the specific surgical context [2, 3].

Deep sedation anesthesia (DSA), also known as monitored anesthesia care (MAC) or intravenous sedation, is an alternative to general anesthesia wherein the patient is deeply sedated but not entirely unconscious. Septoplasty can be performed under deep sedation anesthesia in combination with local anesthesia. The primary advantage of deep sedation anesthesia is a faster recovery time and fewer complications compared to general anesthesia. However, the drawbacks include the possibility of the patient being aware of the surgery and experiencing discomfort or pain. Additionally, providers administering deep sedation must be able to recognize when a patient has entered a state of general anesthesia and maintain their vital functions until they return to the appropriate level of sedation [4, 5].

Thus, nasal septal surgery can be performed under general anesthesia or deep sedation anesthesia. General anesthesia offers a deeper level of unconsciousness and better control of the patient’s vital signs, but it may involve a longer recovery time and higher risk of complications. Conversely, deep sedation anesthesia provides faster recovery and fewer complications, but it may necessitate the provider to manage the patient’s level of sedation and consciousness more closely. The choice of anesthesia depends on the patient’s medical history, the surgeon’s expertise, the specific requirements of the procedure, and the complexity of the surgery. In our study, we considered these surgical parameters in our analysis, recognizing that they can significantly influence the choice of anesthesia and the surgical outcomes.

In the present manuscript, the results of a retrospective study comparing the outcomes of patients who underwent procedures with general anesthesia versus those with sedation will be shown. By employing a propensity score matching model, the researchers aimed to address potential biases and confounding factors in the comparison. This approach allows for a more robust analysis of the effectiveness, safety, and other relevant factors associated with the two anesthesia methods in a real-world clinical setting.

## Methods

### Study design

Following the STROBE protocol, the present study is a retrospective, single-center, non-profit observational analysis that collects data from patients who underwent rhino septoplasty procedures under general anesthesia and sedation at the Santo Stefano Hospital in Prato in a day surgery setting from January 1st, 2017 to December 31st, 2021. Ethical approval for this study was given by Comitato Etico Regione Toscana–Area Vasta Centro, Florence, Italy, regional Institutional Review Board (IRB), (Ethical Committee number: 22659_OSS) on February 21st 2023 (Chairperson Prof. Marco Matucci Cerinic). Written informed consent was obtained from all participants prior to their inclusion in the study.

Patients who meet the following inclusion criteria were considered eligible:

- Inclusion criteria:
  - Age between 18 and 80 years
  - ASA I and II
  - Informed consent
- Exclusion criteria:
  - Age below 18 or above 80 years
  - Prediction of difficulty in intubation (EL-Ganzouri Score higher than 4)
  - Presence of liver and/or renal insufficiency
  - Epilepsy
  - Positive history of allergy to the drugs used
  - Expected surgical intervention lasting more than 1 h
  - Chronic obstructive pulmonary disease
  - NYHA class III and IV
  - History of ischemic heart disease
  - History of arrhythmia

Variables:

From the analysis of medical records, the extracted data will include:

- Age (years)
- Gender (M or F)
- Body weight (kg)
- Height (cm)
- BMI (kg/m^2^)
- Start time of anesthesia (hh:mm)
- End time of anesthesia (hh:mm)
- Duration (hh:mm)
- Average BIS (Bispectral Index)
- Procedure recall (yes or no)
- Pain (VAS - Visual Analog Scale)
- Aldrete 9 recovery time (hh:mm)
- Time of discharge to the ward (hh:mm)
- Time of hospital discharge (hh:mm)
- Average TCI (target controlled infusion) propofol marsh model value (mcg/ml)
- Average dosage of remifentanil in continuous infusion (mcg/kg/min)

### Data collection methods

Upon the patient’s entrance into the operating room, the following parameters were monitored:

- Blood pressure
- Continuous ECG
- Peripheral saturation
- Bispectral Index (BIS) as neurological monitoring of anesthesia depth
- End tidal CO2 (ETCO2)

Patients who underwent rhino septoplasty with sedation received pantoprazole 40 mg IV and dexamethasone 4 mg IV before the surgical procedure to ensure gastric protection and reduce the risk of postoperative nausea and vomiting (PONV). An intravenous infusion of remifentanil at 0.025 mcg/kg/min and 2% propofol was initiated using the target-controlled infusion (TCI) methodology according to the Marsh model, achieving a CSE (concentration at the effector site) of 1.5–2.0 mcg/mL. The Bispectral Index (BIS) was used to evaluate the appropriate level of sedation and was maintained between 65 and 70 throughout the surgical procedure. To prevent closure of the glottis due to tongue obstruction, a Guedel airway was placed, and additional oxygen was administered via nasal cannula at 5 L/min inside the Guedel airway.

Before performing a bilateral infraorbital nerve block (using the intrabuccal technique) and local anesthesia of the nasal mucosa with a mixture of 0.75% naropine and 1:200,000 adrenaline for a total of 20 mL, local anesthesia with 2% lidocaine spray was administered to the nasal mucosa. In case of intraoperative changes in hemodynamic parameters with an increase in heart rate and blood pressure of 10% from baseline values, remifentanil titration occurred by modifying 0.1 mcg/mL using the target-controlled infusion (TCI) system.

Ten minutes before the end of the surgical procedure, paracetamol 1 g IV, ketorolac 30 mg IV, betamethasone 4 mg IV, and ondansetron 4 mg IV were administered. At the end of the surgical procedure, the propofol infusion was stopped, and the infusion rate of remifentanil was gradually reduced until the patient fully regained consciousness.

In patients undergoing rhinoplasty under general anesthesia, preoperative pantoprazole 40 mg IV and dexamethasone 4 mg IV were administered for gastric protection and prevention of postoperative nausea and vomiting (PONV). An intravenous infusion of remifentanil at 0.025 mcg/kg/min and 2% propofol using the TCI method based on the Marsh model was initiated to achieve a CSE of 2.5–3.0 mcg/mL, with neurological monitoring through BIS maintained between 40 and 60 throughout the surgical procedure. Orotracheal intubation was performed after administering 0.3 mg/kg of rocuronium. Bilateral infraorbital nerve blocks were performed using an intraoral technique, followed by local anesthesia of the nasal mucosa with 0.75% naropine and 1:200,000 adrenaline for a total of 20 mL.

If there were intraoperative changes in hemodynamic parameters, with a 10% increase in heart rate and blood pressure from baseline values, remifentanil titration occurred by modifying 0.1 mcg/mL using the Target-Controlled Infusion (TCI) system. Ten minutes before the end of the surgical procedure, 1 g of IV paracetamol, 30 mg of IV ketorolac, 4 mg of IV betamethasone, and 4 mg of IV ondansetron were administered. At the end of the surgical procedure, the propofol infusion was stopped, and the remifentanil infusion was gradually reduced until discontinuation, followed by the administration of 2 mg/kg of sugammadex for neuromuscular blockade reversal. Once the patient was fully awake and cooperative, extubation was performed. In the event of intraoperative bleeding, general anesthesia was induced, and the patient was intubated to ensure airway protection.

For postoperative analgesia, 1 packet of oral paracetamol and codeine (500 mg/30 mg) was administered every 8 h for 5 days following the surgical procedure.

The use of a large language models (LLMs) was carried out in order to edit the present manuscript.

### Data analysis

#### Statistical analysis

The statistical analysis was conducted using R software (the R Foundation, Vienna, Austria). We made use of various R packages, including MatchIt for propensity score matching, WeightIt for inverse probability weighting, and R’s base package for linear regression.

Continuous variables were represented as mean and standard deviation, while categorical ones were expressed as frequencies and percentages. To compare continuous and categorical variables, we employed the independent *t* test or Mann–Whitney test and the chi-square test or Fisher exact test, respectively. A *p* value less than 0.05 was deemed significant. For handling missing data, we used imputation, defaulting to the mean of observed values.

Our study harnessed the power of propensity score matching (PSM) and inverse probability weighting (IPW) to equilibrate the characteristics of patients undergoing either deep sedation anesthesia or general anesthesia. The propensity scores were derived from a logistic regression model, which considered pivotal covariates linked to both anesthesia type and outcome. The MatchIt package enabled us to apply PSM, forming matched patient pairs across anesthesia groups. On the other hand, WeightIt was instrumental in achieving IPW, helping to re-weight observed data and create a pseudo-population with balanced covariate distributions.

Post this matching and weighting procedure, we assessed covariate balance between the groups, resorting to standardized mean differences and visual inspections of propensity score distributions. Sensitivity analyses were pivotal in deciding the most apt model for our study, focusing on balancing covariates, bias reduction, and precision optimization. Our chosen model excelled in equilibrating covariates and producing unbiased treatment effect estimates.

We further employed a linear regression model to ascertain the relationship between anesthesia type and discharge time, our primary outcome. Before proceeding with the linear regression, we ensured all foundational assumptions were met and addressed any outliers or influential points. The regression was executed using the lm function in R, with subsequent diagnostics and validations ensuring model adequacy and reliability.

#### Sample size calculation

Considering that the average postoperative hospital stay time before discharge was 159 ± 65 min for patients undergoing sedation, while those undergoing general anesthesia had a stay time of 318 ± 64 min, an effect size of 0.75 was estimated. Using G*Power software version 3.1.9.7, with a power of 95% and a type I error of 0.001 for a Wilcoxon Mann–Whitney test comparing two means, the required number of cases and controls was estimated to be 12 with a 1:1 ratio.

## Results

**Fig. 1:**
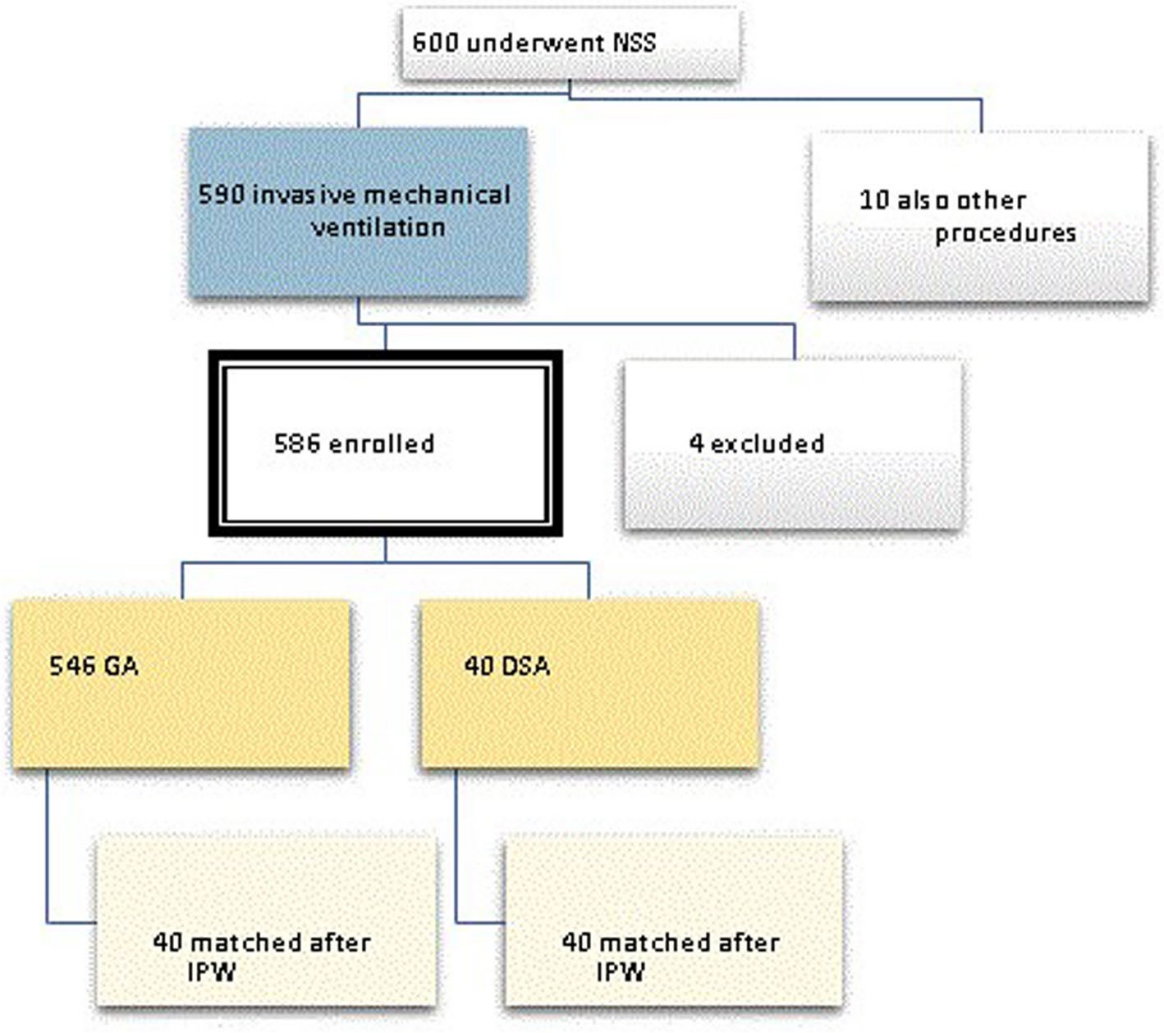
Flowchart of the study (NSS = nasal septal surgery, GA = general anesthesia, DSA = deep sedation anesthesia), IPW = inverse probability weighting)

**Fig. 2:**
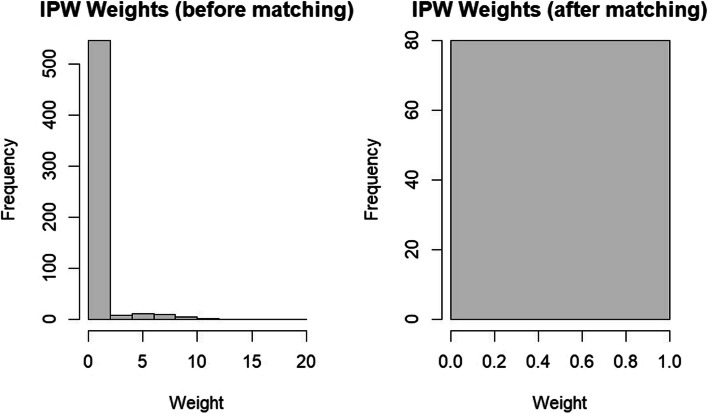
Distribution of IPW before and after matching

From January 1, 2017, to December 31, 2021, a total of 586 participants who underwent rhino septoplasty in the otolaryngology operating rooms of Santo Stefano Hospital in Prato, Italy, were enrolled. Of these, 40 underwent deep sedation analgesia (DSA), while the remaining participants received general anesthesia. Additionally, considering the significant disparity between those who underwent DSA and those who received general anesthesia, both a propensity score matching (PSM) model and an inverse probability weighting (IPW) model were applied. Considering that the IPW sensitivity is lower than PSM and that not all participants were matched in the PSM dataset, it was decided to use the IPW dataset for statistical analysis (Fig. 1). The choice of IPW over PSM in this context may be more appropriate due to its ability to handle the entire dataset, as opposed to PSM, which may result in some unmatched participants being excluded from the analysis. Additionally, the lower sensitivity value for IPW could indicate a more conservative estimate of the treatment effect, which may be preferable in certain research contexts (Fig. 2).

Patients’ characteristics are shown in Table 1.

**Table 1:** Patients’ characteristics

	DSA	GA
Age	40.73 ± 13.82	36.40 ± 11.76
Sex male	20	20
Height	169.57 ± 6.95	175.45 ± 11.49
Weight	66.20 ± 11.93	67.45 ± 11.73
BMI	22.89 ± 2.78	22.82 ± 6.89
LOS_HOS	0	0.88 ± 0.56
Time of anesthesia	53.50 ± 23.18	67.58 ± 13.53
Time of surgery	44.27 ± 22.58	52.58 ± 13.73

*BMI* body mass index, *LOS_HOS* length of stay hospital

**Fig. 3:**
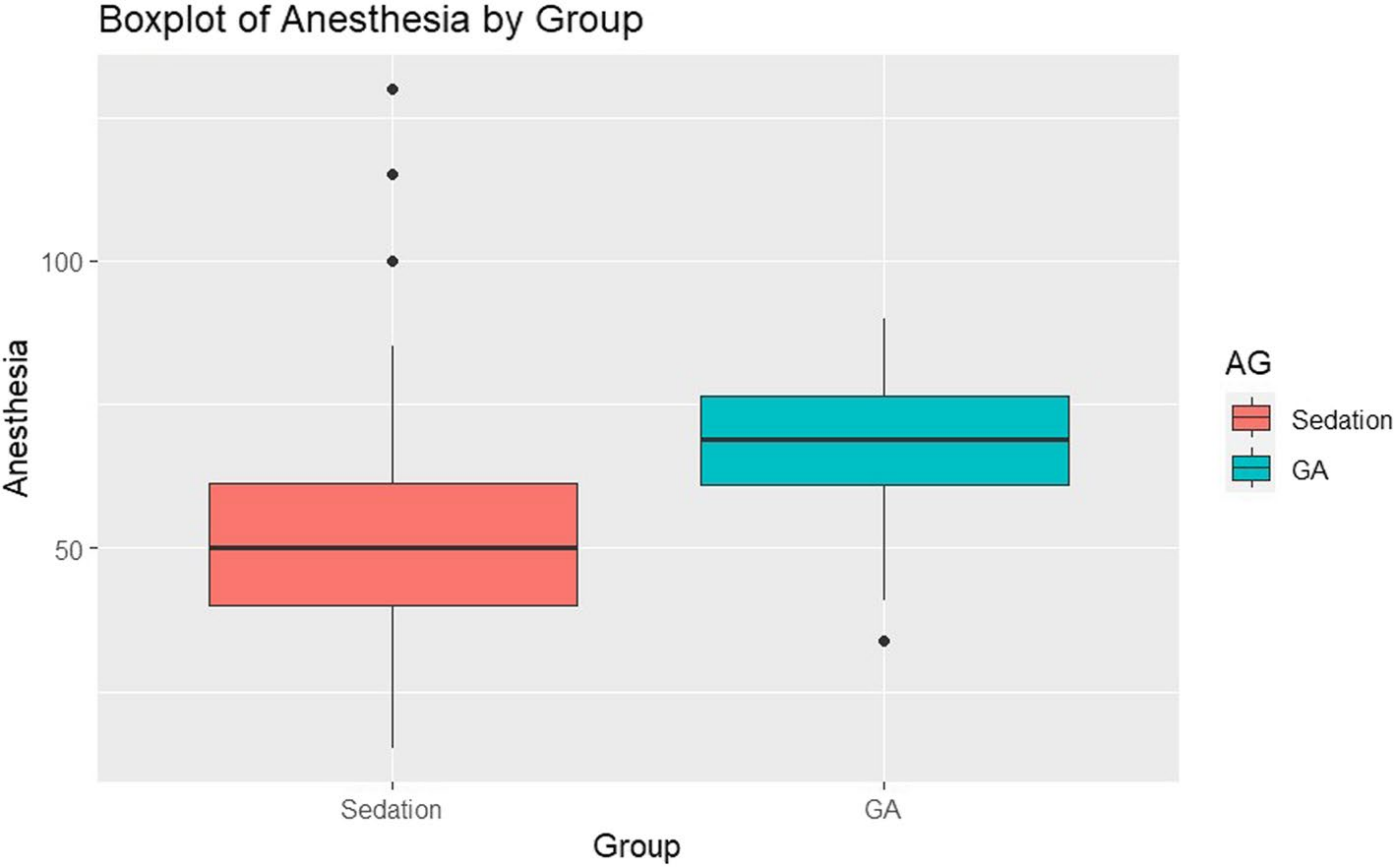
Boxplot of duration of anesthesia in both groups

Upon confirming the non-normal distribution of the data using the Shapiro–Wilk test, a Wilcoxon rank-sum test was conducted for the analysis. Regarding the duration of anesthesia, it was found to be shorter for those who received DSA compared to general anesthesia, with *p* < 0.001 (Fig. 3).

**Fig. 4:**
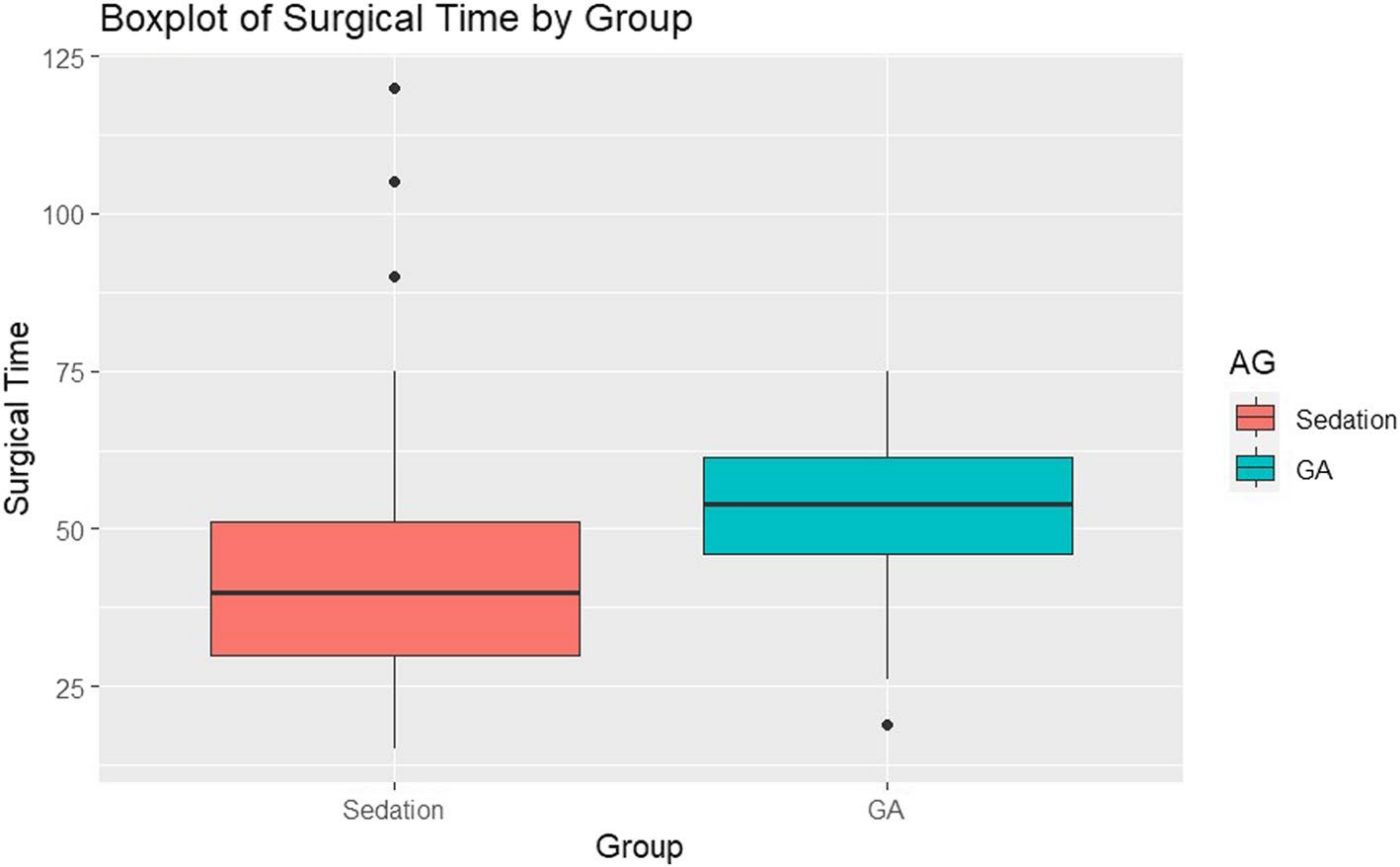
Surgical time in both groups

However, no significant differences were observed in the duration of the surgical procedure between the two groups considered (Fig. 4).

Then it was computed a multivariate linear regression model, which estimates the relationship between the dependent variable Discharge time (expressed in minutes of stay in hospital) and the two independent variables Anesthesia time and type of Anesthesia, based on the matched data.

**Fig. 5:**
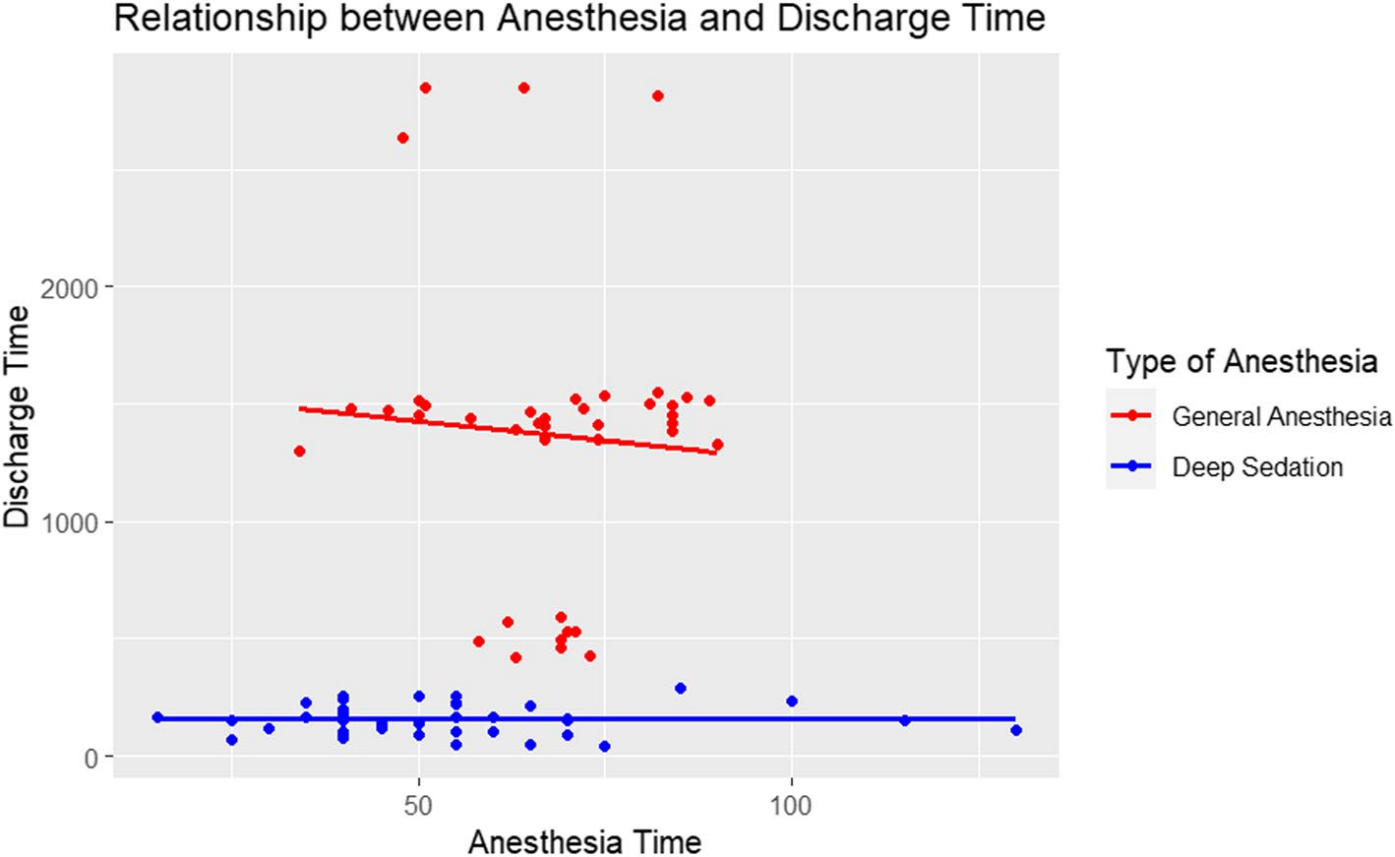
Scatterplot for multivariate linear model for discharge time, anesthesia time and type of anesthesia

The intercept term represents the estimated discharge time value when both anesthesia and AG are zero. The coefficient for anesthesia time indicates the change in discharge for a one-unit increase in anesthesia time, holding type of anesthesia constant. The coefficient for type of Anesthesia represents the estimated difference in discharge between the two levels of Anesthesia (DSA or GA), when Anesthesia time is held constant. In this model the adjusted *R*-squared value of 0.6477 indicates that the model explains 64.77% of the variance in discharge time, after adjusting for the number of variables in the model. The *F*-statistic tests the overall significance of the model, and the very low *p* value (< 2.2e − 16) indicates that the model is highly significant. Overall, the model suggests that type of anesthesia has a strong positive association with Discharge time, while Anesthesia time has a weaker negative association, although not statistically significant (Fig. 5).

The intercept term in our regression model represents the estimated discharge time value when both Anesthesia and AG (type of anesthesia) are set to zero. The coefficient for anesthesia time portrays the change in discharge time for every one-unit increment in anesthesia time, while keeping the type of anesthesia constant. The coefficient linked with the type of anesthesia signifies the estimated disparity in discharge time between the two levels of Anesthesia (DSA or GA), given that anesthesia time remains constant.

Initially, our model had an adjusted R-squared value of 0.6477, suggesting that it elucidated 64.77% of the variance in Discharge Time, when accounting for the number of variables. The *F*-statistic evaluated the overall significance of the model, and the extremely low *p* value (< 2.2e − 16) confirmed the model’s high statistical significance. The primary takeaway was that the type of anesthesia bore a potent positive relation with discharge time, whereas anesthesia time exhibited a more subdued negative relation, albeit not achieving statistical significance (Fig. 5).

**Fig. 6:**
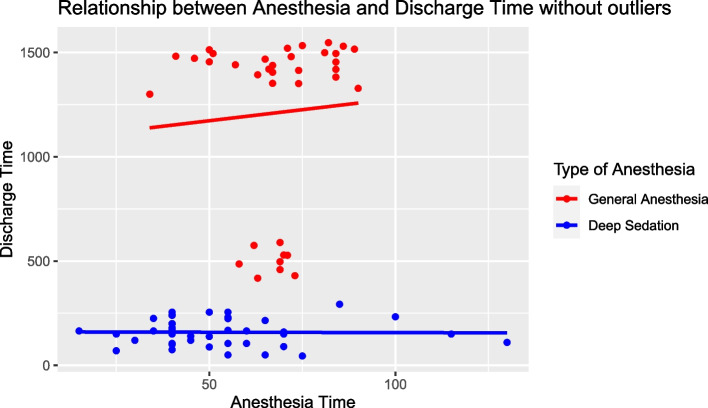
Scatterplot for multivariate linear model for discharge time, anesthesia time and type of anesthesia of filtered data after Cook’s distance

However, we revisited our data and utilized Cook’s distance to identify and omit influential points. This refinement led to a heightened model fit, with the adjusted R-squared value surging to 0.7613. This implies that the revised model, after accounting for influential outliers, now captures roughly 76.13% of the variance in discharge time. Despite this enhancement, the relationship between anesthesia time and discharge time remained statistically non-significant, yet the impact of the type of anesthesia on discharge time persisted in its statistical significance (Fig. 6).

Upon further examination of the data, we conducted an additional analysis focusing on two specific groups:

- A cluster of GA patients with a discharge time of less than 750 min.
- Patients who did not receive GA.

The intercept value was found to be 160.836, signifying the estimated discharge time when both anesthesia time and type of anesthesia were zero. This value was statistically significant (*p* value 1.53e − 07). The coefficient for Anesthesia Time was − 0.03946, indicating a slight decrease in discharge time for every unit increase in anesthesia time, though this relationship was not statistically significant (*p* value 0.93). The type of anesthesia (GA or not) was a significant predictor of discharge time. Specifically, patients under general anesthesia from the identified cluster exhibited a discharge time that was, on average, 343.03428 units longer than their counterparts who underwent deep sedation anesthesia. This relationship was highly significant (*p* value < 2e − 16).

In terms of model fit, the adjusted *R*-squared value was 0.8082, suggesting that this model explained approximately 80.82% of the variance in discharge time for these two groups. The overall significance of the model was confirmed with an *F*-statistic value of 102.2 (*p* value < 2.2e − 16).

**Fig. 7:**
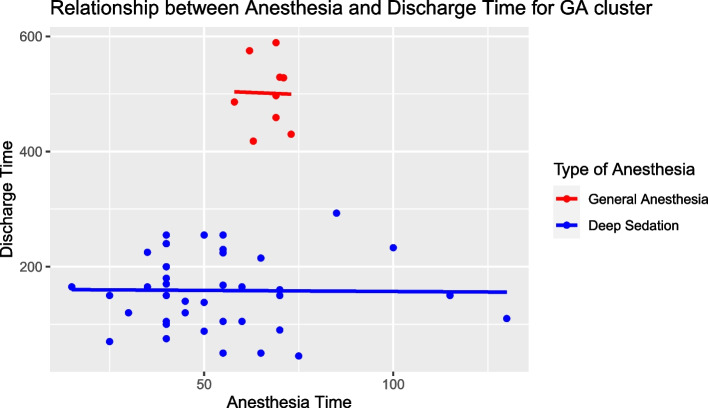
Scatterplot for multivariate linear model for discharge time, anesthesia time, and type of anesthesia of filtered data after clustering GA discharge time < 750 min

This enhanced analysis further underscores the importance of the type of anesthesia in influencing discharge time, especially when considering the specific group of GA patients within the identified cluster (Fig. 7).

## Discussion

In this study, during DSA, a shorter duration of anesthesia time was demonstrated compared to GA in patients undergoing nasal septal surgery. Additionally, we discuss how sedation anesthesia makes it more feasible to perform nasal septal surgery as an outpatient procedure compared to general anesthesia.

Propensity score matching and inverse probability weighting are statistical techniques used to reduce bias in observational studies by matching treated and control subjects based on their probability of being treated, considering their observed characteristics [6, 7]. In this study, the treatment groups were patients who received deep sedation anesthesia and those who received general anesthesia. By employing propensity score matching, we aimed to create comparable groups that allowed for a more accurate comparison of the outcomes of the two anesthesia methods.

The results of our study revealed that deep sedation anesthesia was associated with a significantly shorter anesthesia time compared to general anesthesia. This finding aligns with previous research suggesting that deep sedation anesthesia generally has a faster recovery time and fewer complications than general anesthesia [5]. A shorter anesthesia duration can lead to several benefits for both the patient and the healthcare system. For patients, a shorter anesthesia time may result in fewer side effects, reduced risk of complications, and faster recovery. For the healthcare system, shorter anesthesia times can lead to increased efficiency, reduced costs, and the possibility of performing more procedures within the same timeframe.

One crucial implication of our study’s findings is that using sedation anesthesia for nasal septal surgery could make it more suitable for outpatient procedures compared to general anesthesia. Outpatient procedures, also known as ambulatory or same-day surgeries, allow patients to return home on the same day as the surgery, which can offer several advantages to both patients and healthcare providers. Some of these benefits include reduced hospital stays, lower infection rates, increased patient satisfaction, and cost savings.

In the context of nasal septal surgery, the shorter anesthesia time associated with deep sedation anesthesia can facilitate faster postoperative recovery, making it more feasible to discharge patients on the same day of the surgery. This is particularly important as nasal septal surgery is a common procedure that can benefit from the efficiencies of outpatient care. Additionally, with the growing trend towards outpatient surgeries in various medical fields, it is essential to explore and adopt anesthesia techniques that support this approach. The choice between deep sedation and general anesthesia is not solely an anesthetic decision but a collaborative one. Both the anesthesiologist and the surgeon must be willing and prepared to manage the chosen method. The anesthesiologist must ensure the safety and comfort of the patient, while the surgeon must operate efficiently within the constraints of the chosen anesthesia. This collaboration ensures the procedure’s success and the patient’s well-being. Furthermore, the anticipated surgical field can greatly influence the choice of anesthesia. Factors such as the complexity of the deviation, expected surgical duration, and potential complications can steer the decision towards one method over the other. It is imperative to consider these factors and discuss them thoroughly during the pre-operative planning to ensure the best outcomes.

However, it is essential to consider that sedation anesthesia may not be suitable for all patients or all types of nasal septal surgery. The choice of anesthesia should be tailored to each patient’s medical history, the surgeon’s expertise, and the specific requirements of the procedure. For example, patients with certain medical conditions or those undergoing more complex surgeries may still require general anesthesia to ensure their safety and comfort during the operation. A possible alternative combining is general anesthesia with infratrochlear nerve block/pre-emptive infiltration to achieve a good analgesia plan assuring at the same time the comfort of GA [8, 9].

While our study has demonstrated the benefits of deep sedation anesthesia in terms of anesthesia time and outpatient feasibility, it is important to acknowledge the limitations of retrospective studies and propensity score matching. Although propensity score matching helps to reduce bias and confounding factors, it cannot entirely eliminate them. There may still be unobserved factors that influence the outcomes of the two anesthesia methods. Therefore, future prospective studies or randomized controlled trials could provide more robust evidence to support our findings.

Our study suggests that using sedation anesthesia could potentially streamline nasal septal surgeries, making them more manageable as outpatient procedures. This approach could offer benefits to both patients and healthcare providers. However, it is important to remember that the choice of anesthesia should always be tailored to the individual patient’s needs and the specific requirements of the surgery. While our initial findings are promising, they need to be reinforced by additional research, including prospective studies and randomized controlled trials. The decision on anesthesia is not just about time and cost. Patient comfort, surgeon preference, and other clinical factors play crucial roles. Future studies should delve deeper into these aspects to provide a more comprehensive understanding of what influences the choice of anesthesia for septoplasty. While our findings suggest potential advantages of sedation anesthesia, we recognize that in the USA, most septoplasty procedures are outpatient surgeries, regardless of the anesthesia type. Therefore, our findings might be more relevant in settings where inpatient septoplasty is more common. More research is needed to explore the potential benefits of sedation anesthesia in the context of outpatient septoplasty.

## Data Availability

The data used in this project is available to requesters upon formal request. Requests should be made in writing and sent to the data owner at the following address: rianimazione.ss@uslcentro.toscana.it. All requests will be reviewed on a case-by-case basis, and data access will be granted only if the requester meets the criteria for access and the data are not subject to any legal or ethical restrictions. Access to the data may require the execution of a data sharing agreement, which will outline the terms and conditions of data use, including confidentiality and appropriate use. Any fees associated with the provision of the data will be communicated to the requester in advance of data access.
